# TRP Channels Regulation of Rho GTPases in Brain Context and Diseases

**DOI:** 10.3389/fcell.2020.582975

**Published:** 2020-11-10

**Authors:** Boris Lavanderos, Ian Silva, Pablo Cruz, Octavio Orellana-Serradell, María Paz Saldías, Oscar Cerda

**Affiliations:** ^1^Program of Cellular and Molecular Biology, Institute of Biomedical Sciences, Faculty of Medicine, Universidad de Chile, Santiago, Chile; ^2^Millennium Nucleus of Ion Channel-Associated Diseases (MiNICAD), Santiago, Chile; ^3^The Wound Repair, Treatment and Health (WoRTH) Initiative, Santiago, Chile

**Keywords:** TRP channels, Rho GTPases, actin cytoskeleton, TRP interactome, GEFs, GAPs

## Abstract

Neurological and neuropsychiatric disorders are mediated by several pathophysiological mechanisms, including developmental and degenerative abnormalities caused primarily by disturbances in cell migration, structural plasticity of the synapse, and blood-vessel barrier function. In this context, critical pathways involved in the pathogenesis of these diseases are related to structural, scaffolding, and enzymatic activity-bearing proteins, which participate in Ca^2+^- and Ras Homologs (Rho) GTPases-mediated signaling. Rho GTPases are GDP/GTP binding proteins that regulate the cytoskeletal structure, cellular protrusion, and migration. These proteins cycle between GTP-bound (active) and GDP-bound (inactive) states due to their intrinsic GTPase activity and their dynamic regulation by GEFs, GAPs, and GDIs. One of the most important upstream inputs that modulate Rho GTPases activity is Ca^2+^ signaling, positioning ion channels as pivotal molecular entities for Rho GTPases regulation. Multiple non-selective cationic channels belonging to the Transient Receptor Potential (TRP) family participate in cytoskeletal-dependent processes through Ca^2+^-mediated modulation of Rho GTPases. Moreover, these ion channels have a role in several neuropathological events such as neuronal cell death, brain tumor progression and strokes. Although Rho GTPases-dependent pathways have been extensively studied, how they converge with TRP channels in the development or progression of neuropathologies is poorly understood. Herein, we review recent evidence and insights that link TRP channels activity to downstream Rho GTPase signaling or modulation. Moreover, using the TRIP database, we establish associations between possible mediators of Rho GTPase signaling with TRP ion channels. As such, we propose mechanisms that might explain the TRP-dependent modulation of Rho GTPases as possible pathways participating in the emergence or maintenance of neuropathological conditions.

## Introduction

Neurological diseases encompass broad pathophysiological events, characterized by alterations such as hypo- or hyper-connectivity of synapses, tumoral growth, and loss of neuronal networks (Forrest et al., 2018; La Rosa et al., 2020; Liu Y. et al., 2020). Nevertheless, in most of the cases the precise cellular and molecular mechanisms involved in the pathogenesis of these disorders have not been fully described. Ion channels have been widely described as pivotal molecular entities that contribute to brain physiology and are associated to the development of various brain diseases, thus emerging as highly potential pharmacological targets (Bagal et al., 2013). Several members of the TRP ion channel family play key roles in the regulation of cellular and tissue structures, such as membrane protrusions, synapses, endothelial barriers and glial architecture (Goswami and Hucho, 2007; Goswami et al., 2007a; Gorse et al., 2018; Zhao et al., 2018; Cornillot et al., 2019), whose abnormal activity is intimately linked to neurodevelopmental and neurodegenerative disorders (Morelli et al., 2013). Despite that, how TRP channels participate in brain physiology and brain-affecting diseases is still not fully understood. The concerted activity of Rho GTPases has been described as a signaling node that governs structural maintenance and dynamic changes of cell architecture, mainly through the control and integration of cytoskeleton rearrangements and membrane trafficking. Similarly to TRP channels, Rho GTPases command neurophysiological processes such as dendritic spines morphology, axon cone growth, glial cells migration, blood-brain barrier permeability and vascular tone (Wu et al., 2005; Stankiewicz and Linseman, 2014; Pennucci et al., 2019). Thus, coordinated TRP channels and Rho GTPases activity might be a conserved mechanism for the modulation of changes in physiological and pathophysiological contexts.

### Rho GTPases in Actin-Based Processes in Brain

**FIGURE 1:**
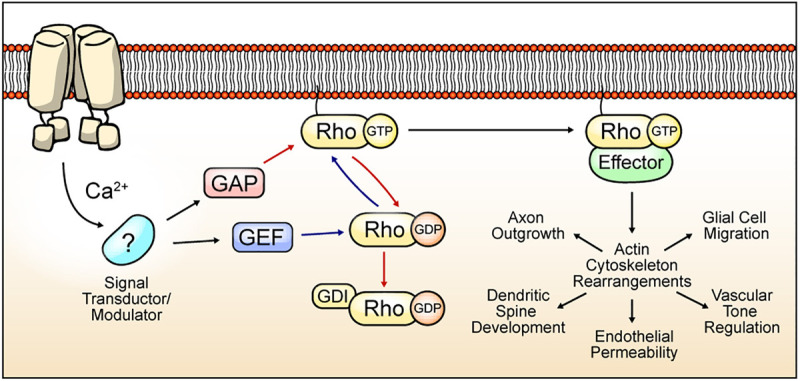
Cyclic Rho GTPase modulation, functions, and TRP channel-mediated regulation hypothesis. Rho GTPases cycle between an active form bound to GTP (blue arrows) and an inactive form that is attached to GDP (red arrows). Binding to guanosine nucleotide is induced by GEF proteins. GAP proteins promote GTPase activity, and GDI proteins preclude GDP exchange to GTP. The active state of Rho GTPase promotes the rearrangement of the actin cytoskeleton by the modulation of several effectors. The actin cytoskeleton mediates multiple processes depending on the cell types. In neurons, axon outgrowth and dendritic spine dynamics regulation. In brain vasculature, Rho GTPases regulate endothelial permeability and vascular tone regulation. Rho GTPases regulates migration in glial cells. TRP channels-mediated Ca^2+^ influx regulates signal transductors/modulators, leading to changes in GAP, GEF, or GDI activity, thus promoting Rho GTPases activation/inactivation and actin cytoskeleton regulation.

Ras Homologs (Rho) proteins are a family of small GTPases belonging to the Ras superfamily. Since the discovery of the first Rho protein (Madaule and Axel, 1985), twenty members have been identified in mammals, currently grouped in 8 subfamilies (Vega and Ridley, 2008). These proteins cycle between an “*active”* GTP-bound state, and an “*inactive”* GDP-bound state. The basis for the cyclic activity of Rho GTPases lies in 3 regulators: (1) Guanine Exchange Factor (GEF) proteins, which catalyze swapping of GDP to GTP, allowing the transition of Rho proteins into their *“active”* state; (2) GTPases-Activating Proteins (GAP), which increase the intrinsic GTPase activity of the guanosine nucleotide-binding protein, promoting GTP hydrolysis and leading to the *“inactive”* state (Bos et al., 2007); and (3) Guanine Dissociation Inhibitors (GDI), which sequester the inactive form of Rho GTPases to prevent their activation by GEFs (Dovas and Couchman, 2005) (Figure 1).

Rho GTPases are crucial for the regulation of the actin cytoskeleton (Rottner et al., 2017) and processes involving cellular motility such as contractility, migration, and membrane protrusions (Machacek et al., 2009). RhoA, Rac1 and Cdc42 are the most studied and distinguished members of this family (Ridley and Hall, 1992; Ridley et al., 1992; Wedlich-Soldner et al., 2003). In neurons, actin cytoskeleton remodeling is essential for several processes including neuronal growth (Marsh and Letourneau, 1984), dendrite development and synapse formation (Sin et al., 2002). These events are indispensable for proper establishment and plasticity of brain circuits. Thus, neurons require accurate actin cytoskeleton regulation, which in turn depends on the fine spatiotemporal and coordinated control of Rho GTPases activity (Murakoshi et al., 2011). Accordingly, the modulation of Rho GTPases has been described to occur in a time/length scale of minutes/micrometers (Pertz, 2010). Canonically, Rac1 activation facilitates the formation of dendritic spines (Luo et al., 1996) and membrane ruffles, while RhoA inhibits these processes and promotes spine shortening (Tashiro, 2000). Moreover, while Glutamate-promoted Cdc42 activity is confined to stimulated dendritic spines, active RhoA spreads along the dendrites (Murakoshi et al., 2011). Thus, spatiotemporal regulation of Rho GTPases is a key point in spine structural plasticity (Hedrick et al., 2016). In addition to their role in neurons, Rho GTPases also play pivotal functions on brain vessels and glial cells. For instance, Rho GTPases regulate contractility and permeability of endothelial brain vessels (Kutcher et al., 2007; Kruse et al., 2019), as well as coordinate different morphological changes on glial cells (reviewed in Zeug et al., 2018). Therefore, these proteins are an essential component for maintaining brain homeostasis and deregulation of their activity lead to several neurological pathologies (Huang et al., 2017).

The precise coordination of Rho GTPases activity highly depends on GEFs, GDIs and GAPs defined by establishing differential protein–protein interactions (PPIs) of Rho GTPases and their modulators. For instance, Rac1 regulation is mediated by selective association with other proteins such as Tiam1 (Rac-GEF), Bcr (Rac-GAP) (Um et al., 2014), and Vilse (Rac-GAP) (Lim et al., 2014). Moreover, RhoA activity is regulated by the interaction of Graf1c (RhoA-GAP) with the tyrosine kinase Pyk2 in postsynaptic neurons, resulting in spine retraction (Lee et al., 2019). Furthermore, Rho GEFs that contain the PDZ-domain interact with lysophosphatidic acid, which promotes RhoA activation (Yamada et al., 2005). Hence, GAP and GEF differential localization and/or interactions are crucial elements for the spatiotemporal regulation of Rho GTPases. Consistently, mutations on these proteins (GAP/GEF) lead to several congenital malformations or developmental neuropathologies (O’Brien et al., 2000; Bai et al., 2015; Pengelly et al., 2016). In this context, the existence of over 70 GEFs and over 70 GAPs adds a further level of complexity in the regulation of Rho GTPases (Müller et al., 2020). In line with this, GEFs and GAPs are tightly regulated by protein interaction partners, second messengers such as Ca^2+^, and posttranslational modifications (PTM) such as phosphorylations (Bos et al., 2007) (Figure 1).

Rho GTPases are also modulated by posttranslational modifications (PTMs). Prenylation of the carboxyl-terminal is the most frequent modification, which entails the addition of a 15- or 20- carbon isoprenoid to a cysteine residue immersed in a *CAAX* motif (Roberts et al., 2008; Reddy et al., 2020). This PTM causes Rho GTPase targeting to the plasma membrane (Hodge and Ridley, 2016), leading to GTP-bound Rho GTPases interaction with their effectors and regulators (Reddy et al., 2020). Moreover, several Rho GTPases phosphorylations, ubiquitylation, sumoylation, and their differential upstream enzymes and outcomes have been reported (extensively reviewed in Hodge and Ridley, 2016).

The activity of several Rho GTPases is linked to depolarization and Ca^2+^ influx in neurons (Hirata et al., 1992; Szászi et al., 2005). This Ca^2+^ influx is related to Voltage-gated Ca^2+^ Channels (VGCCs), although intracellular Ca^2+^ reservoirs and Store-Operated Ca^2+^ Entry (SOCE) also might have an important role in the activation of Rho GTPases as has been widely reported (Jin et al., 2005; Saneyoshi and Hayashi, 2012; Bollimuntha et al., 2017). In this context, most of the mechanisms described involved in Ca^2+^-dependent Rho GTPase modulation rely on Calmodulin (CaM) and Calmodulin-dependent Kinases (CaMKs) as transductors of Ca^2+^ signals through the regulation of GEFs, GAPs, and GDIs. For example, neurotrophins induce intracellular long-range Ca^2+^ waves, increasing RhoA activity during axon formation (Takano et al., 2017). Interestingly, Ca^2+^ waves promote CaMKI activation, leading to recruitment and interaction with GEF-H1, promoting RhoA activation (Takano et al., 2017). Also, CaMKII phosphorylates GEFs and GAPs of Rac1 and Cdc42, such as the Rac1 GEF Tiam1, and RICS, a Cdc42/Rac1 GAP (Okabe et al., 2003). CaMKII-dependent phosphorylation of the Rac1 GEF Kalirin-7 promotes cytoskeleton rearrangement and leads to the plasticity of dendritic spines (Xie et al., 2007).

In this context, the correlation between local/broad Ca^2+^ signals and the activation of different Rho GTPases is an interesting issue, but the molecular entities responsible for these Ca^2+^ signals have not been fully identified. TRP channels are implicated in a myriad of cellular processes associated with actin-based events in migratory cells, through the regulation of Rho GTPases activity (Canales et al., 2019). These channels also play an important role in brain physiology (Dussor et al., 2014; Zeng et al., 2016). As such, TRP channels might serve as key hubs for signaling transduction related to Rho GTPases activities, regulating several features of brain function and architecture.

## TRP Channels

The TRP ion channel family comprises six subfamilies of non-selective cationic channels in mammals, corresponding to TRPA, TRPC, TRPM, TRPML, TRPP, and TRPV (Clapham et al., 2001). All members present six transmembrane domains (S1 – S6) and cytoplasmic amino- and carboxyl-terminal segments of varying length, and a pore-loop between S5 and S6 domains (Table 1) (Hellmich and Gaudet, 2014). The first identified TRP channel in mammals was named as classical or canonical (TRPC). The remaining subfamilies have been named depending on the designation of the first identified member of the respective subfamily, which relies on activation properties, function and/or featured domains (Montell, 2005; Venkatachalam and Montell, 2007).

**TABLE 1:** TRPA, TRPC, TRPV, TRPM and TRPML channels features.

**TRP subfamily**	**Size**	**P_*Ca*_^2+^_/_P_*Na*_^+^**	**Domains N-term**	**Domains C-term**	**Phosphoinositides-Binding**	**Localization**
**TRPA**	∼1000 aa	0.8–1.4	Ankyrin-repeats	TRP-box-like motif Inositol phosphate binding region Coiled-coil region	PIP_2_ – Activation	Plasma Membrane
**TRPC**	<1000 aa	0.5 – 9	Ankyrin-repeats Coiled-coil regions	TRP-box motif Calmodulin-binding CIRB domains PDZ domains	PIP_2_-Activation/Inhibition	Plasma Membrane
**TRPV**	<900 aa	1 - >100	Ankyrin-repeats	TRP-box motif Calmodulin-binding	PIP_2_ – Activation/Inhibition PG- Activation (V1) PI- Activation (V1) PS- Activation (V1)	Plasma Membrane
**TRPM**	<1500 aa	0.5–10 (Except M4/5 which have <0.05)	Melastatin family channel homology region	TRP-box motif Coiled-Coil region Nudix (M2) Kinase (M6/7)	PIP_2_ – Activation	Plasma Membrane Melanosomes (M1)
**TRPML**	<600 aa	∼1	–	EF-Hand motifs	PIP_2_ – Activation	Endolysosome vesicles

Transient receptor potential channels can be activated by multiple stimuli, such as temperature, pH changes, and membrane mechanical stress (Clapham, 2003). Moreover, the activity of these channels can be modulated by PIP_2_ (Nilius et al., 2008; Rohacs, 2014), PTMs such as phosphorylation (Cerda et al., 2015; Xu et al., 2019; Liu X. et al., 2020) and interacting proteins (Singh et al., 2002; Zhu, 2005; Cho et al., 2014; Rivas et al., 2020). Monomers can form heterotetrameric functional channels with distinctive properties in relation with their homotetrameric counterparts (Chubanov et al., 2004; Ma et al., 2011; Kim et al., 2019). Heteromultimerization can occur not only among members of the same TRP subfamily but also with those of different subfamilies (Ma et al., 2011). These features and the wide expression of these channels in multiple tissues and cell types, grant a high level of complexity to their role in diverse physiological and pathophysiological processes (Venkatachalam and Montell, 2007). In this context, several TRP channels are implicated in cellular processes related to central nervous system (CNS) functioning such as the modulation of neuronal excitability (Mickle et al., 2016; Hong et al., 2020b), maturation or establishment of subcellular structures such as dendrites (Tai et al., 2008), excitatory synapses (Zhou et al., 2008), axonal outgrowth (Jang et al., 2014), frontal cortex postnatal development (Riquelme et al., 2018) and brain blood flow regulation (Earley et al., 2004; Cornillot et al., 2019). Also, current evidence suggests that TRP channels could regulate downstream processes such as gene expression or more lasting effects, like Long Term Potentiation (LTP). Hence, it is not surprising to find CNS pathophysiological conditions associated with TRP channels activity (Table 2). TRP channels have been associated to stroke (Zhang and Liao, 2015), CNS ischemia-reperfusion damage (Gauden et al., 2007; Chen et al., 2017; Leiva-Salcedo et al., 2017), status epilepticus (Kim et al., 2013; Phelan et al., 2017), Alzheimer’s and Parkinson’s diseases (Zhang et al., 2013; Hong et al., 2020b), and progression of neoplasms of neuronal (Chen et al., 2014; Middelbeek et al., 2015) or glial (Lepannetier et al., 2016; Ou-Yang et al., 2018) origin.

**TABLE 2:** TRP channels-associated pathologies and possible Rho GTPases-dependent mechanisms.

**TRP Channel**	**TRP Channel activity deregulation-associated disease**	**Possible Rho GTPase-dependent mechanism for TRP channel role in disease**
TRPC3	Mwk phenotype (Wu et al., 2019), Cerebellar ataxia (Dulneva et al., 2015)	Aberrant spine remodeling/morphology due to deregulation of CaMKII/Tiam1 axis
TRPC5	Substance abuse (Pomrenze et al., 2013) – Excitotoxicity (Phelan et al., 2013)	Rac1 and DR-2-mediated regulation of RhoA
TRPC6	Glioma (Ding et al., 2010). AD (Lessard et al., 2005; Feng, 2017)	Glioma: Promotion of FAK activation, cell proliferation and migration
		AD: Loss of TRPC6 expression leads to the activation of Rac1, promoting the amyloidogenic pathway
TRPV1	AD (Balleza-Tapia et al., 2018; Du et al., 2020), Glioblastoma (Nabissi et al., 2016)	AD: Protective effect due to induction of axonal filopodia or dendritic spines Glioblastoma:Increment of cell invasion through RhoA activity
TRPV2	Possible participation in neurodegenerative processes	Rac1/RhoA-mediated neuritogenesis and tubulogenesis modulation
TRPV4	ICH, TBI (Zhao et al., 2018), AD (Zhang et al., 2013)	RhoA-mediated endothelial dilation and vascular tone regulation
	Glioma progression (Ou-Yang et al., 2018)	Increased cell migration due to enhanced Rac1 activity
TRPM4	IS (Leiva-Salcedo et al., 2017; Chen et al., 2019), glutamatergic toxicity (Schattling et al., 2012), SCI (Gerzanich et al., 2009)	Rac1-mediated induction of NADPH oxidase and ROS production
TRPM7	IS (Chen et al., 2015), Neuroblastoma (Middelbeek et al., 2015)	IS: RhoA upregulation and subsequent induction of cell death.
		Neuroblastoma: RhoA-dependent cytoskeleton remodeling Endothelial Barrier disruption
TRPM8	Migraines (Gavva et al., 2019; Ling et al., 2019), Glioblastoma (Zeng et al., 2019)	Regulation of cerebral arterial vasodilation
TRPML1	Mucolipidosis type IV (Bargal et al., 2000)	Control of Rho GTPases-dependent membrane trafficking

*ICH, intracerebral hemorrage; IS, ischemic stroke; TBI, traumatic brain injury; SCI, spinal cord injury; AD, Alzheimer’s disease.*

**FIGURE 2:**
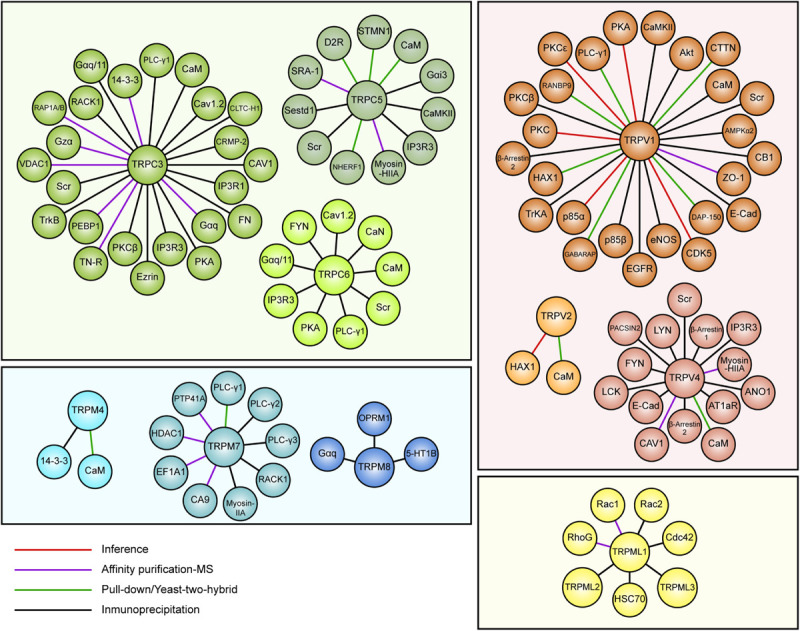
Map of curated TRP ion channel interactors associated with Rho GTPases pathways. Data obtained from the TRIP database showing interactors of TRPC3, TRPC5, TRPC6, TRPV1, TRPV2, TRPV4, TRPM4, TRPM7, TRPM8, and TRPML1. TRP channels members are grouped and color-coded by families.

Several reports suggest a role for TRP channels on Rho GTPases regulation. In this context, interactome maps based on different Protein–Protein Interaction databases suggest associations between TRP channels with Rho GTPases-related proteins. For instance, the TRIP database, a curated database from individual studies that report protein interactions with TRP channels, provides insightful information regarding the TRP-Rho GTPase interactions (Shin et al., 2011). Other general interactome databases such as BioGRID (Oughtred et al., 2019) and BioPlex (Schweppe et al., 2018), generated from high-throughput interactome datasets and curated individual studies, complement these interactions. The resulting interactome map reveals that the TRP-interacting proteins network reveals several Rho GTPases-related regulators, such as Src, PLC-γ1, Akt, PKC and the GTPase Gαq (Shin et al., 2011) (Figure 2). In this context, the activity or expression of TRPC3 (Kitajima et al., 2011; Numaga-Tomita et al., 2016), TRPC5 (Tian et al., 2010) TRPC6 (Singh et al., 2007; Tian et al., 2010), TRPM4 (Cáceres et al., 2015), TRPM7 (Su et al., 2011), TRPV1 (Li J. et al., 2015; Li et al., 2015a), and TRPV4 (Ou-Yang et al., 2018; Zhao et al., 2018) increase the activity of several Rho GTPases (Table 3). Also, there is evidence showing that TRPM8 (Sun et al., 2014), TRPV2 (Laragione et al., 2019), and TRPV4 (Thoppil et al., 2016) channels inhibit Rho GTPases (Table 3). These opposite effects could rely on multiple mechanisms that give versatility to Ca^2+^ signals-dependent responses such as specific localization in membrane subdomains or cellular substructures of the channels, leading to differential and dynamic interactions with Ca^2+^-regulated proteins that might modulate Rho GTPases. This regulation might occur *via* activation of Ca^2+^-dependent kinases proteins such as PKC and CaM kinases, leading to direct regulatory phosphorylations on Rho GTPases (Hodge and Ridley, 2016). Despite the above, the elevated number of GAPs and GEFs (Müller et al., 2020) and the diverse outcomes on cytoskeleton rearrangement elicited by TRP channels (Kuipers et al., 2012; Canales et al., 2019) might point to an indirect TRP channels-dependent regulation of Rho GTPases through these modulators (GEFs and GAPs).

**TABLE 3:** Rho GTPases regulated by TRP channels.

**TRP Channel**	**Effect on Rho GTPase Regulation**	**Downstream Rho GTPase**	**Possible mechanism for GTPase modulation by TRP channel**
TRPC3	Positive	Rac1 (Kitajima et al., 2011)	CaMKII-induced Rac1 GEF Tiam1 phosphorylation/activation*
TRPC5	Positive	Rac1 (Tian et al., 2010)	Interaction with SESTD1*
	Negative	Rhoa A (Tian et al., 2010)	Rac1 positive modulation, leading to RhoA inhibition*
TRPC6	Positive	RhoA (Singh et al., 2007; Tian et al., 2010; Jiang et al., 2011)	Calcineurin-dependent regulation of RhoA*
	Negative	Rac1 (Tian et al., 2010)	Downregulation of Rac1 GEF Tiam1 by TRPC6-mediated NMDA receptor inhibition*
TRPV1	Positive	RhoA (Li J. et al., 2015; Li et al., 2015a)	TRPV1-mediated Inhibition of AMPA receptor endocytosis*
TRPV2	Negative	Rac1/RhoA (Laragione et al., 2019)	PKA activation by TRPV2-driven cAMP increases, leading to RhoA inhibition*
TRPV4	Positive	RhoA (Zhao et al., 2018)	PKC-dependent RhoA activation (Brain endothelial cells, Zhao et al., 2018)
		Rac1 (Ou-Yang et al., 2018)	Increasing AKT phosphorylation levels (U87 glioma cells, Ou-Yang et al., 2018)
TRPM4	Positive	Rac1 (Cáceres et al., 2015)	Modulation of Ca^2+^ signals – Vm depolarization-dependent phosphatidylserine translocation*
TRPM7	Positive	RhoA, Rac1, CDC42 (Su et al., 2011)	RhoA: Modulation by p116RIP* Rac1: S1P1 activation through Mg^2+^ influx (Endothelial cells [Zhu et al., 2019])
TRPM8	Negative	RhoA (Sun et al., 2014)	Regulation of mitochondrial function and ROS production, leading to modulation of RhoA/ROCK (Vascular Smooth Muscle cells, Xiong et al., 2017)
TRPML1	Undetermined	Possible modulation of Rac1, Rac2, Cdc42 and RhoG	Interaction with Rac1, Rac2, Cdc42 and RhoG*

*Proposed mechanisms.

In the following sections, based on established and inferred interactions, we will discuss and hypothesize possible mechanisms for TRP channels participation *via* Rho GTPases in different neuropathologies.

### TRPC Channels

TRPC is the founding subfamily of the mammalian TRP channel family, since the first TRP channel found in mammals was named “canonical” owing to its similarities to the TRP channel from *Drosophila* (Montell and Rubin, 1989; Clapham et al., 2001). The mammalian TRPC subfamily can be divided into two subgroups based on the percentage of identity between their sequences: (1) TRPC1/TRPC2/TRPC4/TRPC6 and (2) TRPC3/TRPC6/TRPC7. Of note, TRPC2 is a pseudogene in *Homo sapiens* (Wang et al., 2020). These channels can heteromultimerize with members of the corresponding (TRPC) or other sub-families, which yields functional non-selective ion channels with a wide range of relative P_*Ca*_/P_*Na*_ (Table 1) (Chen et al., 2020). All these channels are responsive to GPCR/RTK-induced PLC activation (Wang et al., 2020). Some members also respond to ER-Ca^2+^ stores depletion *via* STIM1, such as TRPC1, TRPC3, TRPC4, and TRPC5 (Yuan et al., 2007; Lee et al., 2014). Moreover, TRPC channels activity is regulated by PIP(4,5)_2_ and PIP_2_-derivates such as DAG, IP3, PI(4)P and PI. Structurally, TRPC channels are composed by intracellular N-terminal and C-terminal domains, and six membrane-spanning domains. Expression of TRPC channels has been reported in various tissues, such as kidney, salivary glands, hippocampus, pancreatic β cells, heart, and vascular smooth muscle, and therefore participate in a wide variety of physiological processes (Chen et al., 2020).

### TRPC3

TRPC3 channels are characterized by their coupling to tyrosine kinase and G protein coupled receptors activation, acting as mediators of Ca^2+^ signals induced by these receptors (Ambudkar and Ong, 2007). TRPC3 channels are especially abundant in the brain, mainly in the cerebellum, caudate nucleus, putamen and striatum (Riccio et al., 2002). These channels participate in Purkinje cells physiology in the cerebellum (Hartmann et al., 2008). Indeed, mGluR1 promotes TRPC3-dependent rises of local Ca^2+^ signals, leading to slow membrane depolarization at Purkinje cells dendrites (Hartmann et al., 2008). Accordingly, changes in the expression or mutations of TRPC3 gene cause detrimental consequences on motor functions such as the ‘Moonwalker’ (Mwk) phenotype (Wu et al., 2019). Mice with Mwk phenotypes display gait and limb incoordination (Becker, 2017). Interestingly, TRPC3 upregulation in Mwk animals impairs the development of dendrites (Becker et al., 2009). Furthermore, the mutant variant R672H of the human TRPC3 gene leads to cerebellar ataxia (Dulneva et al., 2015). Consequently, TRPC3 activity deregulation might induce structural alterations during the progress of cerebellar ataxia by modulating dendrite development.

**FIGURE 3:**
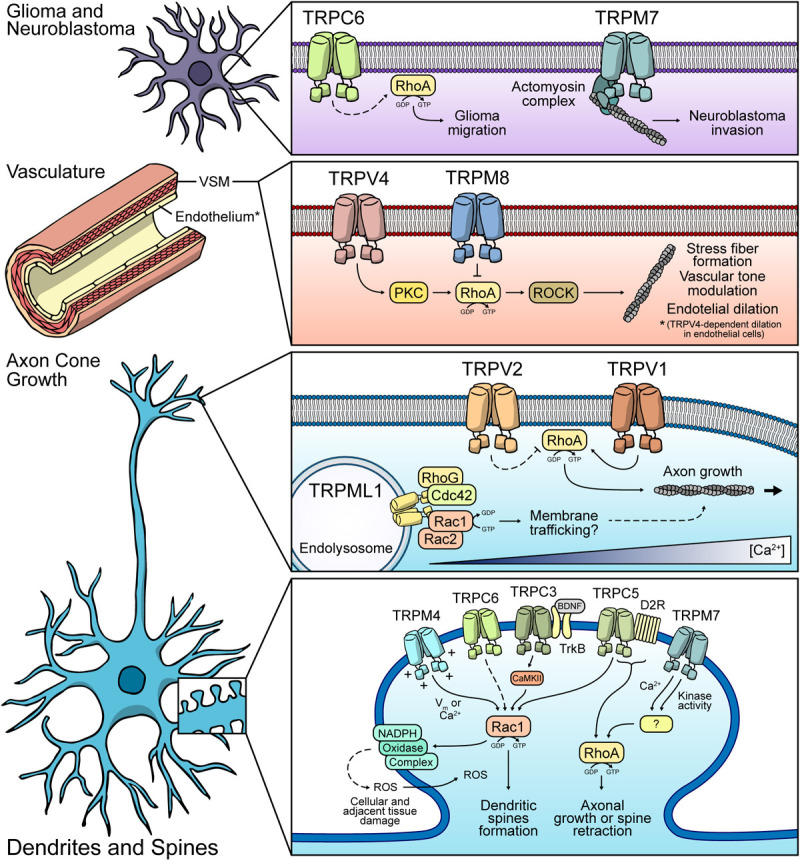
Possible mechanisms of Rho GTPases modulation by TRP channels. Glia and Neuroblastoma: TRPC6 might modulate RhoA in glial cells, thus regulating cell migration. TRPM7 participates in neuroblastoma invasion. Vasculature: TRPV4 and TRPM8 display the opposite effect over RhoA. Both channels modulate vasculature tone. TRPV4 promotes endothelial dilation in a RhoA-dependent fashion. Axon cone: TRPML1-dependent regulation of protein trafficking *via* modulation of Rac1/Cdc42 might occur. TRPV1 promotes RhoA activity and modulates axonal growth. TRPV2 might have an inhibitory role of RhoA. Dendrites and spines: TRPM4, TRPC3, TRPC6, and TRPC5 might induce Rac1 activity, leading to dendritic spines formation. Under ischemic conditions, these channels might have a Rac1-dependent role in ROS production, leading to neuronal cell death. TRPM7 and TRPC5 are proposed to regulate RhoA, favoring the retraction of dendritic spines or axonal growth.

There is not a direct evidence for TRPC3-mediated regulation of Rho GTPases in neurons or brain tissues. Nevertheless, data obtained from other models, as well as TRPC3/Rho GTPases associated pathways, might suggest a functional relationship between these proteins. For instance, BDNF induces Rac1 and Cdc42 activation, but not RhoA, through activation of TrkB receptor (Hedrick et al., 2016). Moreover, BDNF-induced TrkB activation leads to TRPC3 activation, which is necessary to induce spine remodeling (Amaral and Pozzo-Miller, 2007), suggesting that TRPC3 activation might be a mediator for BDNF-dependent activation of Rac1 and Cdc42, although further studies would be needed to confirm this. Interestingly, TRPC3 inhibition in heart mouse model induces a reduction of CaMKII and Rac1 activity (Kitajima et al., 2011). CaMKII-mediated Tiam1 phosphorylation leads in turn to Rac1 activation (Fleming et al., 1999; Buchanan et al., 2000; Tolias et al., 2005). These data suggest that TRPC3 activation by BDNF might induce Tiam1 CaMKII-dependent phosphorylation (Table 3), resulting in dendritic spine remodeling by Rac1 activity (Figure 3). Furthermore, downstream effectors might include PAK1, since its activation by Rac1 that leads to actin remodeling through LIMK1 activity (Edwards et al., 1999). Likewise, CaMKII-ß regulates dendritic spine formation in Purkinje cells through a mechanism that involves mGluR1 and PKC activation. This CaMKII-ß-mediated effect requires IP_3_R activation (Sugawara et al., 2017). Moreover, CaMKII has an important role in the production of LTP by promoting the insertion of AMPA receptors in the post synaptic region (Lisman et al., 2012). Importantly, our curated search in the TRIP database showed that TRPC3 interacts physically and functionally with the IP_3_R isoforms (Kim J.Y. et al., 2006), and also with BDNF receptor TrkB (Li et al., 1999) (Figure 2). Thus, we propose that TRPC3 activation by BDNF/TrkB or mGluR1 in Purkinje might elicit spine remodeling through CaMKIIß activation and subsequent Rac1 activation. This proposed pathway could be relevant for pathologies that entail degeneration of neuronal processes. For example, morphology of dendritic spines is heavily altered in conditions of aberrant CaMKII/Tiam1/Rac1 activity, which might collaborate to intellectual disability of patients bearing mutations of the ATRX-encoding gene (Shioda et al., 2011). These data is consistent with a possible role for TRPC3 in this pathology due to the above-mentioned role of this channel in BDNF-dependent spine remodeling, which might open new avenues of studies for novel TRPC3 functions in brain pathophysiology.

### TRPC5

TRPC5 is another non-selective cation channel that belongs to the TRPC subfamily. TRPC5 activity is related to many sensorial features such as touch and hearing (Sexton et al., 2016), satiety sensation (Gao et al., 2017), and internal physiological pH chemosensitivity (Cui et al., 2011). TRPC5 regulates neurites length (Greka et al., 2003), neurite outgrowth during neuron differentiation (Heo et al., 2012), neurite retraction (Hui et al., 2006), and axonal outgrowth (Oda et al., 2020). Moreover, several studies link the function of TRPC5 to dynamic behavioral processes that are tightly related to structural changes in synapsis, such as fear (Riccio et al., 2009), addictive behavior (Pomrenze et al., 2013), and tolerance to opioids (Chu et al., 2020) (Table 2). Thus, TRPC5 activity has been intimately associated to LTP modulation (Phelan et al., 2013), suggesting a participation on synaptic plasticity and in turn, on brain physiology and pathophysiology. The morphological changes mediated by TRPC5 overlap with Rho GTPases-dependent signaling, since this has also been related to structural modifications required for memory and learning (Diana et al., 2007). Thus, TRPC5 might promote structural dynamic changes through the modulation of Rho GTPases. Interestingly, enhanced Rac1 activity has been reported upon TRPC5 overexpression (Tian et al., 2010), although, the underlying mechanisms remain unclear. Interestingly, TRPC5 interacts with Rho GTPases modulators, such as SESTD1 (Figure 2) (Miehe et al., 2010). SESTD1 is a putative scaffold protein that diminishes dendritic spine density by abolishing the interaction between Rac1 and the Rac-GEF Trio8 (Lee C.C. et al., 2015). Nevertheless, it is not known whether this mechanism could affect the retraction or extension of dendritic spines. In this context, we suggest that SESTD1 interaction with TRPC5 might decrease its available levels to block the Rac1-Trio8 association, causing an indirect TRPC5-dependent increased Rac1 activity. This is consistent with the negative regulatory effect of TRPC5 on neurites growth as discussed above. Rac1 activity promotes the growth of neurites (Woo and Gomez, 2006). Therefore, a possible negative effect of TRPC5 upon neurite extension through Rac1 modulation seems paradoxical. However, it has been recently reported that either hypo or hyperactivity of Rac1 could lead to neuritogenesis impairment, giving arise to intellectual disability (Zamboni et al., 2018). On the other hand, TRPC5 interacts with CaMKII (Puram et al., 2011). This could be relevant since CaMKII is an activator of RhoA and Cdc42, leading to the induction of dendrites growth (Murakoshi et al., 2011). Consistently, TRPC5 promotes dendrite growth through CaMKII activity (He et al., 2012), suggesting that TRPC5 might control this process through CaMKII/RhoA/Cdc42 axis. As mentioned before, CaMKII is important for the production of LTP response (Lisman et al., 2012), which suggests a TRPC5 participation in processes related to memory and learning.

TRPC5 activity is also associated with cell viability. These channels have been proposed to exert either a protective role (Hong et al., 2020a) or promote neuronal loss depending on its PTM status in neurodegenerative disorders such as Huntington’s disease (Hong et al., 2015). Moreover, TRPC5 promotes cell death induced by oxidative stress (Park et al., 2019) and excitotoxicity during epileptic seizures (Phelan et al., 2013). Dopamine D2 receptor (D2R) activity has been reported to enhance cell vulnerability by inducing neurite retraction and axon collapse *via* RhoA/ROCK activity (Deyts et al., 2009). TRPC5 interacts with D2R (Figure 2) (Hannan et al., 2008) and as such, TRPC5 channels might induce cell death in a RhoA-dependent manner, although this still needs to be determined. Interestingly, Rac1 activity is associated with cell death promotion during oxidative stress caused by neurovascular ischemia (Smith et al., 2017; Karabiyik et al., 2018). Moreover, Rac1 activity might also have a protective role in epileptic excitotoxicity (Posada-Duque et al., 2015). In contrast, RhoA activation has been linked to Ca^2+^-dependent neuronal loss elicited by excitotoxicity (Semenova et al., 2007). This dichotomy between protective and degenerative roles of Rho GTPases upon ischemic injury has been widely reviewed elsewhere (Posada-Duque et al., 2014). As such, TRPC5 might have a dual participation in both dynamics of protrusive processes and neuronal death. These effects might depend on differential TRPC5-dependent spatiotemporal regulation of Rho GTPases, a process that could be further analyzed with new tools to study more exactly ion channel-related spatiotemporal events.

### TRPC6

TRPC6 shares close sequence and structural homology with TRPC3. TRPC6 can also be modulated by tyrosine kinase receptors and G protein-coupled receptors, being especially sensitive to activation by diacylglycerol (DAG) (Hofmann et al., 1999). TRPC6 is widely expressed in the peripheral nervous system (Riccio et al., 2002). TRPC6 is located in excitatory post-synaptic neurons and regulates the number of spines and spatial learning and memory (Zhou et al., 2008) *via* CaMKIV activation and CREB regulation, promoting both neurite and dendrite growth (Tai et al., 2008; Heiser et al., 2013). The transcription factor CREB is associated with the late phase of LTP. Its activation results in changes in gene expression that promote synapsis development and structural rearrangements (Frey et al., 1993; Nguyen et al., 1994). This suggests that TRPC6 contributes to the late phase of LTP. Interestingly, CaMKIV has been associated with actin cytoskeleton remodeling, since it regulates LIM Kinase 1 (LIMK1) activity to promote neurite growth (Takemura et al., 2009). In turn, LIMK1 phosphorylated cofilin to favor actin filaments stabilization (Yang et al., 1998). This suggests that TRPC6 activity might affect synapsis formation through the CaMKIV-LIMK1-cofilin pathway. Moreover, several reports suggest a protective role for TRPC6 from excitotoxicity by inhibiting NMDA receptor activity (Li et al., 2012; Shen et al., 2013). Although no direct interaction has been reported between TRPC6 and Rho GTPases, our analysis of the curated list of interactors of TRP channels from the TRIP database (Figure 2) reveals that TRPC6 interacts with several proteins that regulate Rho GTPases function such as Calcineurin (Ly and Cyert, 2017). Furthermore, several studies report the contribution of TRPC6 in RhoA regulation in non-excitable cells (Singh et al., 2007; Tian et al., 2010).

Similarly to TRPC3, TRPC6 has been involved in different brain disorders such as neuronal damage in stroke, Aβ-production in Alzheimer’s disease and especially in the development and progression of glioma (Ding et al., 2010). TRPC6-mediated effects in the brain have been described as a mix between positive and negative. One of the most studied TRPC6-related roles in cerebral pathologies is in the development and progression of gliomas. TRPC6 is overexpressed in glioma samples compared to normal brain tissue. Additionally, its activity is essential for G2/M phase transition and glioma progression (Ding et al., 2010). Moreover, TRPC6 maintains the stability of HIF-1α in glioma cells under hypoxia (Li et al., 2015b) and mediates Notch-Driven glioblastoma growth and invasiveness (Chigurupati et al., 2010). In this context, the RhoA/ROCK signaling pathway is upregulated in glioma cells, promoting cell migration and proliferation (Zohrabian et al., 2009). Interestingly, RhoA/ROCK pathway is activated by TRPC6 in other tissues such as the kidney (Yang et al., 2013), suggesting that this relation between TRPC6 and RhoA/ROCK might also play a role in glioma progression. Moreover, upregulation of TRPC6 and subsequent increased Ca^2+^ influx in podocytes lead to an aberrant activation of focal adhesion kinase (FAK), which is necessary for focal adhesion assembly and disassembly in migratory cells (Nakuluri et al., 2019). RhoA activity can promote the activation of FAK (Al-Koussa et al., 2020), suggesting an additional mechanism for TRPC6-RhoA mediated tumoral progress in glioma (Figure 3). Although there are several possible mechanisms for TRPC6-mediated regulation of the RhoA/Rock pathway in glioma progression, this still needs to be confirmed in the context of this neoplastic disease.

As discussed above, TRPC6 also regulates several beneficial processes in the neurons such as survival, synaptogenesis, learning and memory, all of which are altered in Alzheimer’s Disease (Feng, 2017). Overexpression of presenilin 2 and/or Alzheimer’s-disease-related presenilin 2 variants, decreased TRPC6-mediated Ca^2+^ entry in HEK293 cells (Lessard et al., 2005). Moreover, TRPC6 expression and activity are diminished in neurons from Alzheimer’s-disease patients. Also, pharmacological activation of TRPC6 inhibited the elevation of Aβ and phospho-tau in neurons of patients (Tao et al., 2020), indicating a possible protective role of TRPC6 in Alzheimer’s-disease. Moreover, upregulation of Rac1 appears to be important in driving the progress of Alzheimer’s-disease by promoting the production of the amyloid precursor protein and the amyloidogenic pathway (Aguilar et al., 2017). Importantly, loss of TRPC6 expression leads to the activation of Rac1 in the kidney (Tian et al., 2010), which suggests that the lower expression of this channel observed in the brain of Alzheimer’s-disease patients could also promote the pathology by activating Rac1 (Figure 3).

### TRPV Channels

The TRPV subfamily comprises six members, of which the temperature-sensitive TRPV1 is the founder and the most studied. TRPV1 was first described as an ion channel activated by the vanilloid capsaicin, which explains the name of the subfamily (Caterina et al., 1997, 1999; Güler et al., 2002). The members of the TRPV subfamily share a similar architecture. These channels contain an ankyrin-repeat domain (ARD) and follow six transmembrane helix domains in the amino-terminal region. Cytoplasmatic carboxyl-terminal contains the TRP domain and other binding sites for lipid regulators, all of them with a resolved structure (Yuan, 2019). In addition to the canonical role of TRPV channels in temperature and pain sensing, other functions have been attributed to these channels, wherein the regulation of Rho GTPases emerges. Other characteristics are described in Table 1 and have been extensively summarized previously (Bevan et al., 2014; Garcia-Elias et al., 2014; Kojima and Nagasawa, 2014).

### TRPV1

TRPV1 was the first and most studied member of the vanilloid subfamily of TRP ion channels (Caterina et al., 1997; Benítez-Angeles et al., 2020). TRPV1 can be activated by exogenous agonists such as capsaicin and resiniferatoxin (RTX) (Caterina et al., 1997), as well as high temperature (>42°C), pH and membrane potential changes (Caterina et al., 1997; Piper et al., 1999; Gunthorpe et al., 2000), mechanical forces (Ho et al., 2014), ROS (Starr et al., 2008), arachidonic acid (Chu et al., 2003), and endocannabinoids (Ross, 2003). Initial studies identified TRPV1 in sensory neurons, describing its participation in the regulation of pain transduction. TRPV1 is also expressed in brain cortex, hippocampus, cerebellum, substantia nigra, hypothalamus, midbrain, olfactory bulb and astrocytes (Mezey, 2000; Marinelli et al., 2003; Kim et al., 2005; Kim S.R. et al., 2006; Cavanaugh et al., 2011; Park et al., 2012; Nam et al., 2015). Interestingly, multiple studies associate TRPV1 activity to cytoskeleton remodeling, cellular migration (Ho et al., 2014; Miyake et al., 2015) and cell morphology (Goswami and Hucho, 2007; Goswami et al., 2007b; Li et al., 2015a). For example, mechanical stress-elicited TRPV1-dependent Ca^2+^ signals contribute to astrocyte migration (Ho et al., 2014). Also, mitochondrial TRPV1 enhances microglial cell migration (Miyake et al., 2015). Moreover, TRPV1 localizes in multiple dynamic structures of neurons such as the growth cone, neurites and axonal filopodia, displaying differential roles among these structures. TRPV1 activation results in rapid retraction of growth cones in sensory neurons dependent on microtubule depolymerization (Goswami et al., 2007b). Also, a recent study established that Ca^2+^ influx mediated by TRPV1 is necessary for NGF deprivation-induced axonal degeneration (Johnstone et al., 2019). Interestingly, RhoA activity is necessary for axonal growth cone collapsing and proper polarity establishment for axon guidance (Wu et al., 2005; Loudon et al., 2006). Furthermore, RhoA also controls microtubules protrusion during axonal growth (Dupraz et al., 2019). Importantly, mechanical stress-induced TRPV1 activity participates in guidance during neurite extension of spiral ganglion neurons in a RhoA/ROCK-dependent fashion (Figure 3) (Li et al., 2015a). Thus, along with its participation on microtubule dynamics, TRPV1-dependent modulation of RhoA-ROCK might also cooperate in guidance during axonal growth (Figure 3). Since Ca^2+^ is crucial for axon specification and outgrowth in CNS neurons (Davare et al., 2009; Nakamuta et al., 2011), these TRPV1-dependent proposed effects might occur in dorsal root ganglion (DRG) and CNS neurons as well, although further studies would be needed to confirm this.

TRPV1 regulates axonal filopodia formation, a key process for axonal branching (Spillane and Gallo, 2014). Interestingly, this process is not dependent on the conductive activity of the channel, but only on its C-terminal domain, which interacts with microtubules (Goswami et al., 2007a). TRPV1-induced filopodia resembles those formed upon ROCK and Myosin II inhibition (Loudon et al., 2006). These data contrast with the role of TRPV1 on the regulation of RhoA since this Rho-GTPase canonically inhibits axonal branching (Spillane and Gallo, 2014). We hypothesize that such discrepancy might be due to the structural features rather than the conductive activity of the TRPV1 channels on axon filopodia initiation, leading to a differential mechanism on cytoskeleton regulation.

The complex role of TRPV1 in the modulation of neuronal structures might be involved in neurodegenerative pathologies such as Alzheimer’s disease. Indeed, a protective effect of TRPV1 in Alzheimer’s disease has been reported (Balleza-Tapia et al., 2018; Du et al., 2020). One of the proposed mechanisms consists in TRPV1-mediated inhibition of the AMPA receptor endocytosis, which is a key modulator of spine development *via* GEF-H1 (Kim et al., 2004; Kang et al., 2009). Thus, TRPV1-dependent hampering of AMPAR endocytosis might be a putative mechanism for TRPV1 positive modulation of RhoA, thus contributing to the development of Alzheimer’s disease (Table 3).

TRPV1 has also been proposed as a negative prognosis marker for glioblastoma (Nabissi et al., 2016), in which invasiveness is highly dependent on RhoA activity since it regulates several matrix metalloproteinases in this type of neoplasms (Al-Koussa et al., 2020). Nevertheless, further studies are needed to detail the participation of TRPV1 in the development of neoplastic pathologies associated with neuronal or glial cells in the brain.

### TRPV2

TRPV2 is a member of the vanilloid subfamily of TRP ion channels that is activated by high temperatures (>52°C) (Caterina et al., 1999). TRPV2 is also activated by hypoosmolality (Muraki et al., 2003), cell stretching (Sugio et al., 2017) and chemical stimuli such as 2-aminoethoxydiphenyl borate (2-APB) (Hu et al., 2004), cannabinoids (Qin et al., 2008), and probenecid (Bang et al., 2007). TRPV2 is found in DRG neurons (Caterina et al., 1999), trigeminal motor nucleus (Park et al., 2011), hypothalamus and hindbrain regions (Wainwright et al., 2004; Nedungadi et al., 2012), and astrocytes (Shibasaki et al., 2013). TRPV2 localizes in subcellular domains enriched in filamentous actin, such as the growth cone, filopodia, lamellipodia and neurites, where interacts with soluble actin (Yadav and Goswami, 2019). Furthermore, inhibition of TRPV2 produces retraction of the growth cone (Yadav and Goswami, 2019) (unlike TRPV1), while its activation enhances axon outgrowth (Shibasaki et al., 2010) and growth cone motility (Sugio et al., 2017), along with inducing rapid membrane ruffling, changes in lamellipodial and filopodial dynamics, and rapid translocation of leading edges during neuritogenesis (Yadav and Goswami, 2019). Although the downstream signaling pathway of TRPV2 activity has not been elucidated yet, TRPV2 induces an increase in the cAMP levels in neurites and their branching points (Yadav and Goswami, 2019). TRPV2-dependent increase of cAMP leads to PKA activation, which inhibits RhoA (Figure 3) (Ellerbroek et al., 2003; Oishi et al., 2012). Therefore, the remodeling effect on the actin cytoskeleton by TRPV2 activity might be through modulation of the Rho GTPase family *via* cAMP/PKA (Howe, 2004) (Table 3). Given this data, we hypothesize that these mechanisms might lead to a TRPV2-dependent inhibition of the growth cone retraction. Further studies are needed to determine these mechanisms. Furthermore, PKA activates Rac1 (Goto et al., 2011) and Cdc42 (Leemhuis et al., 2004), crucial proteins for the formation of lamellipodia and filopodia, respectively. Moreover, both GTPases activate PAK1 to promote the axonal growth cone (Toriyama et al., 2013). Thus, these Rho GTPases might also be molecular targets that explain the effects of TRPV2 activity on lamellipodia and filopodia dynamics.

### TRPV4

TRPV4 is a major regulator of muscle- and endothelium-dependent vascular tone in blood vessels from multiple organs such as the intestine, lungs and brain (White et al., 2016). TRPV4 responds to shear stress and lipids, such as arachidonic acid and epoxyeicosatrienoic (EET) acid (White et al., 2016), which are essential vasoactive substances required for vascular dilation/contraction (Sudhahar et al., 2010). The participation of TRPV4 in the modulation of brain vessels is related to the activity of the channel in endothelial cells (White et al., 2016) and vascular smooth muscle cells (Chan et al., 2019). For instance, chronic cerebral hypoperfusion produces a myogenic tone decrease of brain parenchymal arterioles, an effect that was not observed in TRPV4^–/–^ mice (Chan et al., 2019). Additionally, TRPV4 loss-of-function is associated with diminished endothelial dilation, decreased cerebral perfusion and impaired cognitive function in aged rats (Diaz-Otero et al., 2019). Moreover, TRPV4 promotes PKC-dependent RhoA activation, leading to stress fibers formation during internal cerebral hemorrhage (ICH) in endothelial cells (Table 2 and Figure 3), presumably by ROCK activation and MLCP inhibition (Zhao et al., 2018). TRPV4 inhibition ameliorates damage produced by ICH, suggesting that TRPV4 promotes blood brain barrier disruption in ICH conditions by inducing the formation of endothelial intercellular gaps in a way that involves RhoA activation and stress fiber formation (Zhao et al., 2018). Long-range Ca^2+^ signals caused by Ca^2+^-Induced Ca^2+^ Release (CICR) induce RhoA activation after neurotrophin-3 treatment (Takano et al., 2017). TRPV4 promotes CICR in neurons in ICH model, most likely by regulating the IP_3_R (Shen et al., 2019). Moreover, the interplay between TRPV4 and IP_3_R also occurs at astrocytes endfeet, where TRPV4-dependent Ca^2+^ entry induces Ca^2+^ oscillations mediated by IP_3_R, leading to neurovascular coupling and vasodilation (Dunn et al., 2013). In this regard, we propose that the propagation of Ca^2+^ waves in these cells might contribute to RhoA’s spatiotemporal regulation and subsequent cytoskeleton remodeling.

TRPV4 promotes Rac1 activity in glioblastoma U87 cells leading to increased cell migration through TRPV4-dependent Akt phosphorylation/activation (Ou-Yang et al., 2018) (Table 3). Akt activation induces the phosphorylation of the Rac1 GEF Tiam1, which promotes Rac1 activation, driving tumorigenesis (Zhu et al., 2015). In this context, CaMKII has been shown to phosphorylate Akt in ovarian (Gocher et al., 2017) and prostate cancer cells (Schmitt et al., 2012). Thus, local Ca^2+^ signals elicited by these ion channels might promote Ca^2+^/CaM binding to CaMK II, leading to Akt-mediated Rac1 activation through Tiam1, suggesting participation of TRPV4-mediated Rac1 regulation in glioblastoma progression.

### TRPM Channels

Eight members of this subfamily have been identified. These channels have differential ion selectivity between their members. For instance, TRPM4 and TRPM5 are monovalent cationic channels (Launay et al., 2002; Hofmann et al., 2003), while TRPM7 and TRPM8 mediate divalent cationic (Nadler et al., 2001) and non-selective cationic currents (McKemy et al., 2002). TRPM channels share a Melastatin homology region (MHR) domain located at the amino-terminal region, a transmembrane domain similar to other TRP members, and a versatile carboxyl-terminal that varies between the TRPM members. For instance, TRPM7 contains a functional α-kinase domain in the carboxyl-terminal (Nadler et al., 2001), while TRPM4 and TRPM8 possess coiled-coil domains and binding motifs for different regulatory molecules (Fleig and Penner, 2004). The structures of all the TRPM channels discussed in this review have been recently described (Huang et al., 2020). Thus, the TRPM subfamily is a highly versatile subgroup of TRP channels, due to the particular structures and biophysical properties (Fleig and Penner, 2004). TRPM features have been summarized in Table 1 and extensively reviewed (Fleig and Penner, 2004; Huang et al., 2020).

### TRPM4

TRPM4 is activated by intracellular Ca^2+^ increases (Venkatachalam and Montell, 2007), but, unlike other members of the TRP family, TRPM4 (along with TRPM5) is only permeable to monovalent ions. Despite that, TRPM4 regulates Ca^2+^ signals in different cell types, such as fibroblasts (Cáceres et al., 2015), mastocytes (Shimizu et al., 2009), dendritic cells (Barbet et al., 2008), and lymphocytes (Launay et al., 2004). TRPM4 expression has been described in hypothalamus (Teruyama et al., 2011), hippocampal CA1 area (Menigoz et al., 2016), preBötzinger nucleus (Picardo et al., 2019), and medial prefrontal cortex (Riquelme et al., 2018). Interestingly, TRPM4 increases Rac1 activity in MEF cells (Cáceres et al., 2015). Even though, the Ca^2+^-permeable ion channels regulated by TRPM4 that could explain changes in Ca^2+^ oscillations have not been identified yet, functional association between NMDA receptors and TRPM4 in postsynaptic dendrites of CA1 hippocampal neurons has been shown (Menigoz et al., 2016). Indeed, TRPM4 activity-mediated postsynaptic depolarization allows proper lifting of Mg^2+^ block from the NMDA receptor, leading to an increased Ca^2+^ entry and initiation of LTP (Menigoz et al., 2016). Moreover, glutamatergic excitotoxicity is decreased in TRPM4^–/–^ mice, further suggesting a functional interaction between NMDA and TRPM4 (Schattling et al., 2012). Interestingly, Ca^2+^ entry mediated by NMDA receptors, and subsequent CaMKII-dependent Tiam1 phosphorylation, induces dendritic/spine development (Tolias et al., 2005). Thus, interplay between TRPM4 and NMDA receptors might be important in Rac1-dependent neuronal plasticity. Other possible mechanisms for Rac1 regulation by TRPM4 could be the regulation of local changes in membrane potential. For instance, voltage-gated Na^+^ channel Nav1.5 has been proposed to promote Rac1 activity by producing membrane potential depolarization in breast cancer cells (Yang et al., 2020). Nav1.5-induced membrane potential depolarization causes Rac1 activation by promoting local redistribution of phosphatidylserine, an anionic phospholipid to which Rac1 binds, and known to be important for its activation (Finkielstein et al., 2006; Yang et al., 2020). Interestingly, TRPM4 facilitates cellular depolarization in bone marrow-derived mast cells (BMMC) (Vennekens et al., 2007), HeLa (Simon et al., 2010), and HEK293 cells (Fliegert et al., 2007). Thus, local changes in membrane potential mediated by TRPM4 might induce local phospholipid redistribution and Rac1 activation (Figure 3).

TRPM4 participates on damage caused by ischemic stroke (Leiva-Salcedo et al., 2017; Chen et al., 2019) and spinal cord injury (Gerzanich et al., 2009). Importantly, TRPM4 inhibition and silencing improves outcome of both spine cord injury (Gerzanich et al., 2009) and ischemic stroke (Loh et al., 2014; Chen et al., 2019). TRPM4 induces cellular swelling leading to neuronal cell death as a consequence of excessive Na^+^ influx mediated by these channels (Gerzanich et al., 2009; Simon et al., 2010; Leiva-Salcedo et al., 2017). Interestingly, Rac1 participates in NADPH oxidase assembly (Acevedo and González-Billault, 2018). Moreover, increased activity of Rac1 induces ROS production by promoting the activation of the NADPH catalytic subunit, Nox2 (Stankiewicz and Linseman, 2014). This enzyme has been proposed as a potential ischemic stroke therapeutic target (Zhang et al., 2016). Consistently, Rac1 downregulation, similar to TRPM4 inhibition, leads to a protective effect in the hippocampal CA1 region and cortex in ischemia/stroke models (Raz et al., 2010) and after permanent middle cerebral artery occlusion (Karabiyik et al., 2018). Given that TRPM4 activity might lead to an increase in Rac1 activity, we hypothesize that an additional mechanism by which this channel could collaborate with brain and vascular tissue-damaging, alongside oncotic cell death induction, is by promoting an increase in Nox2-dependent ROS production *via* Rac1 activation (Figure 3). Although this mechanism is only inferred, the suggested evidence makes it an interesting possible new approach to understanding TRPM4-mediated GTPase regulation’s role in brain neuropathologies.

### TRPM7

TRPM7 is a member of the TRP family that permeates both Ca^2+^ and Mg^2+^. In addition, this channel has an active kinase domain in their carboxyl-terminal domain (Sun et al., 2015). TRPM7 is widely expressed in the human and mouse brain, with reports in animal models showing equivalent levels in neurons, astrocytes and microglia (Ratnam et al., 2018). TRPM7 localizes in the growth cone of hippocampal neurons, restricting the elongation of primary axons (Turlova et al., 2016). Interestingly, TRPM7 associates with several elements of cytoskeletal structures. For instance, mass spectrometry-based analyses revealed the interaction of TRPM7 with actin and α-actinin-1 (Turlova et al., 2016). Moreover, analysis of the TRPM7-associated interactome in neuroblastoma cells (Figure 2) identified proteins which were mainly related to the actin cytoskeleton, such as Myosin IIA, Drebrin and the Myosin phosphatase Rho-interacting protein (p116RIP) (Middelbeek et al., 2016). p116RIP is a modulator of the RhoA/ROCK axis (Koga and Ikebe, 2005), a key pathway that promotes invasion and metastasis of tumor cells (O’Connor and Chen, 2013). This suggests a role for TRPM7 in aggressive phenotypes in neuroblastoma cells *via* p116RIP-mediated RhoA/ROCK regulation and subsequent changes in cytoskeleton dynamics. Also, a functional coupling between TRPM7 and RhoA activation dependent on the TRPM7 kinase activity on hepatocellular carcinoma has been shown (Voringer et al., 2020). These data support a TRPM7-mediated RhoA activation involvement in diverse processes such as the inhibition of axonal growth cone and the migratory phenotype of brain tumor cells like neuroblastoma (Figure 3).

Several reports suggest that TRPM7 is a key mediator of neuronal death after ischemia/reperfusion episodes. TRPM7 knock-down in CA1 protected neurons from ischemia-induced cell death as well as maintained neuronal morphology and function (Sun et al., 2009). Moreover, TRPM7 blockage by carvacrol prevented brain damage in a mouse hypoxia-ischemia brain injury model (Chen et al., 2015). TRPM7 silencing leads to a decreased activity of RhoA (Su et al., 2011). This might be important in the context of ischemic damage since RhoA is upregulated in the brains of human stroke patients (Brabeck et al., 2003). The RhoA/ROCK pathway is key to determining neuronal cell death following ischemia/reperfusion events (Shin et al., 2008). This suggests that one of the mechanisms by which TRPM7 might be producing its pathological effect after ischemia/reperfusion is through the activation of RhoA. However, further studies into this association will help clarify this suggested relationship and find additional mediators.

Mg^2+^ permeability of TRPM7 is a particularity among the TRP family. A misbalance in the homeostasis of this ion has been involved in several pathological processes in the brain (Sun et al., 2015). TRPM7-mediated increases of Mg^2+^ intracellular concentration promotes endothelial cell proliferation and enhances the endothelial barrier integrity of the brain. This is due to induced cytoskeletal reorganization and expression of tight junction proteins such as VE-cadherin, occludin, and zonula occludens-1 (ZO-1) (Zhu et al., 2019). Interestingly, TRPM7 achieves this through the activation of S1P1, which in turn activates Rac1 (Zhu et al., 2019). This suggests that the TRPM7-mediated Mg^2+^ influx might play an important role in cerebral vasculature pathologies by regulating the S1P1-Rac1 pathway.

### TRPM8

TRPM8 is a non-selective cation channel, which preferably permeates Ca^2+^ (McKemy et al., 2002). TRPM8 has a polymodal gating and is activated by cold and different cooling compounds, such as menthol and icilin (McKemy et al., 2002). Several single nucleotide polymorphisms (SNPs) related to the TRPM8 gene are associated with either the risk (Ling et al., 2019) or protection (Gavva et al., 2019) from migraines. Although the exact mechanisms are unknown, a possible mechanism for TRPM8-mediated risk of migraine headaches might be related to this channel’s role in vasoconstriction regulation, which has been related to migraine development (Jacobs and Dussor, 2016). Interestingly, TRPM8 activation by menthol promoted vasodilation by inhibiting Ca^2+^ signaling–mediated RhoA/ROCK activation in mesenteric arteries (Figure 3) (Sun et al., 2014). In this regard, the RhoA/ROCK axis inhibition might lead to Myosin Light Chain Phosphatase (MLCP) activation and LIM Kinase 1 inhibition, resulting in an inhibition of the contraction of mesenteric arteries (Shimokawa et al., 2016). Furthermore, TRPM8 can function as an ER Ca^2+^ channel, regulating mitochondrial function and ROS production, which activates the RhoA/ROCK pathway (Xiong et al., 2017) (Table 3). Thus, TRPM8-dependent vascular tone modulation through the RhoA/ROCK axis regulation might play an important role. These mechanisms might not only be relevant in the development of migraines but also the progress of other cerebral vasogenic events such as reversible vasoconstriction syndromes.

Recent reports have associated alterations in TRPM8 expression with brain-related cancers. Moreover, TRPM8 was significantly overexpressed in glioblastoma tissue samples compared to normal tissue and its expression correlated with worse prognosis and survival in glioblastoma patients (Zeng et al., 2019). Also, TRPM8 was highly expressed in glioblastoma cell lines and its expression correlated with higher invasive and proliferative capacities (Zeng et al., 2019). In this context, TRPM8-mediated regulation of RhoA/ROCK might be responsible for the pro-tumoral effect of TRPM8, as the RhoA/ROCK pathway has been involved in the proliferation and migration of glioblastoma cells (Fortin Ensign et al., 2013). These data support a role for TRPM8 in diverse aspects of brain pathophysiology, including some that are more particular to this channel. Thus, more studies into the exact proteins mediating TRPM8-mediated RhoA/ROCK activation might be interesting to elucidate its precise role.

### TRPML1

The Mucolipin-TRP (TRPML) subfamily are monovalent and divalent cation channels with predominant Ca^2+^ permeability (Peng et al., 2020) (see Table 1), although other permeabilities has been described to be important in their function (Dong et al., 2008). Contrary to other TRP subfamilies, these channels are particularly located in endolysosomal vesicles (Manzoni et al., 2004). Consistently, TRPML channels are associated with cellular processes such as vesicle trafficking and endolysosomal-dependent degradation pathways (Cheng et al., 2010). Structurally, these channels have full-length subunits of about 600 amino acids, thus being the smaller members of TRP channel subfamily. As to the other TRP channels, these channels have six transmembrane segments with pore-forming regions located between segments S5 and S6. In addition, amino- and carboxyl-terminal regions have cytoplasmatic orientation (Hellmich and Gaudet, 2014). Moreover, these channels have canonical EF-hand domains at their carboxyl-terminal domain, which allows direct Ca^2+^-dependent activity modulation (Hellmich and Gaudet, 2014). TRPML activity is positively modulated by PIP_2_ binding, which drives to channel activation (Dong et al., 2010). Interestingly, the resolved structure of TRPML1 indicates that its unique PIP_2_-binding site is distinct to the reported in others TRP channels (Fine et al., 2018) (Table 1).

TRPML1 (also called Mucolipin-1) is an intracellular TRP channel located predominantly in endolysosomal membranes (Lee J.H. et al., 2015). Loss-of-function mutations reported in the TRPML1-encoding gene have been linked to a lysosomal storage disorder known as Mucolipidosis type IV (MLIV). This pathology is one of the first described neurological diseases caused by mutations in TRP channels (Bargal et al., 2000). MLIV is characterized by an early neurodevelopmental delay and a late neurodegenerative phenotype that leads to intellectual disability, delayed psychomotor abilities and retinal abnormalities (as described in OMIM #252650). Although the pathophysiological signs are well known, the cellular mechanisms involved in this disease remain unclear. TRPML1 modulates several aspects of membrane trafficking, particularly participating in trafficking of late endosome vesicles, and controlling the size (Cao et al., 2017), function and biogenesis of lysosomes (Wang et al., 2015). Moreover, TRPML1 regulates lysosome fusion with secretory vesicles, increasing exocytosis (Park et al., 2016). Interestingly, not only a disturbance in membrane trafficking but also lipid accumulation and loss of cell viability have been described as hallmarks of MLIV disorder (Wang et al., 2013), suggesting that TRPML1 loss-of-function might contribute in all these pathological alterations. However, detailed mechanisms by which TRPML1 regulates these processes are poorly understood. Rho GTPases are signaling nodes that coordinate membrane trafficking and lipid homeostasis (reviewed in Ridley, 2001; Olayioye et al., 2019). TRPML1 interactome reports (Figure 2) might reveal the mechanisms related to the coordination of cellular processes that lead to the neuropathological phenotype observed in MLIV (Krogsaeter et al., 2019). TRPML1 interacts and colocalizes with Rho GTPases, such as Rac1, Rac2, Cdc42 and RhoG (Spooner et al., 2013). Therefore, these data strongly suggest that TRPML1 might regulate the activity of Rho GTPases and, in this manner, control membrane trafficking (Figure 3). Thus, regulating TRPML1 activity might be an effective therapeutic approach for the treatment of other neurological disorders where low TRPML1 activity has been reported, such as ALS (Tedeschi et al., 2019), Parkinson disease (Tsunemi et al., 2019), Alzheimer’s Disease (Zhang et al., 2017), and Niemann-Pick disease (Shen et al., 2012). Nevertheless, further studies are needed to determine the specific role of TRPML1-dependent Rho GTPases modulation in these neuropathological conditions and the proteins involved in this relationship.

## Outstanding Questions and Future Directions

Despite extensive evidence on the role of the canonical Rho proteins (RhoA/Rac1/Cdc42) in different neuronal processes and their possible modulation by TRP channels activity presented in this work, little is known about other Rho GTPase members. A limited number of studies have shown the participation of RhoF/RhoD (RhoF/Rif, RhoD) (Zanata et al., 2002; Fan et al., 2015) and atypical Rho GTPases (Aspenström et al., 2007) such as Rnd (Rnd1, Rnd2, and Rnd3/RhoE) (Ishikawa et al., 2003, 2006; Heng et al., 2008; Pacary et al., 2013), RhoU/RhoV (Wrch1, Chp/Wrch2) (Alan et al., 2018) and RhoBTB (RhoBTB3 and RhoBTB2) (Ramos et al., 2002) in neuronal processes. However, these proteins play an important role in developmental synaptogenic stage (Ishikawa et al., 2003), growth cone turning and collapse (Zanata et al., 2002), neurite formation (Tian et al., 2017) and retraction (Fan et al., 2015), axon guidance (Alan et al., 2018), cortical neuron migration (Heng et al., 2008; Tian et al., 2017), and neurogenesis (Pacary et al., 2013). Although no studies indicating that TRP channels regulate these Rho GTPase subfamilies, it has been reported that NFAT1c increases RND1 transcription (Suehiro et al., 2014) and rapid activation and translocation of NFAT to the nucleus is promoted by TRPV1 activity in sensory neurons (Kim and Usachev, 2009). Conversely, Rif has been established as a regulator of cytoskeletal rearrangements mediated by semaphorins (Tian et al., 2017). TRPC5 acts downstream of semaphorin signaling to cause neuronal growth cone morphology (Kaczmarek et al., 2012). These antecedents suggest that both TRP channels and atypical Rho GTPase activity could be involved in the different processes mentioned above. However, more studies need to be performed.

Regarding the discussion raised in this review, the TRP family of ion channels and Rho GTPases are functionally linked to each other due to; (1) mutual cellular functions whose deregulation leads to neuropathological phenomena and (2) Rho GTPases activity modulation by TRP channels. However, spatiotemporal features of TRP channel activity, and how these features could regulate Rho GTPase are still not clear.

TRP-associated interactome suggests that these channels associate with different Rho GTPases-related proteins (Shin et al., 2011) (Figure 2). This allows us to hypothesize a potential role for TRP channels as transduction hubs, implying local recruitment of Rho GTPases modulators and spatially delimited Rho GTPases activation/inhibition in subcellular structures. Moreover, Rho GTPases are mainly activated by Ca^2+^ signaling in neurons (Saneyoshi and Hayashi, 2012). Ca^2+^-sensitive proteins such as the Ca^2+^/CaM complex play an essential role in Rho GTPase activity modulation (Buchanan et al., 2000; Vidal-Quadras et al., 2011). Differential TRP-CaM binding affinities could lead to subtle changes in the local activity of Ca^2+^/CaM-dependent proteins, such as CaMKs activation and subsequent Rho GTPases modulation (Wayman et al., 2011; Bossuyt and Bers, 2013). Conversely, TRP channels interaction with downstream targets of Rho GTPases suggests that these channels can be regulated by these proteins. Accordingly, Rho GTPases participate in the regulation of TRP channels trafficking and activity (Bezzerides et al., 2004; Puntambekar et al., 2005). Thus, TRP channels can regulate Rho GTPases and *viceversa*, suggesting a regulatory feedback loop between these proteins.

However, our research demonstrates that the relationship between TRP ion channels and Rho GTPases is not always positive (Figure 3). For instance, TRPM8 activation induces RhoA inhibition, while other members like TRPM7 and TRPC5 promote the activity of this Rho GTPase. We reasoned that multiple aspects contribute to establishing the relation between the respective TRP channel and Rho GTPase. Interactome and subcellular localization of both proteins and the biophysics properties of the channel might explain these differences. As we reviewed, most of the TRP channels that regulate Rac1 in neuronal context are localized in the same subcellular compartments (Figure 3).

Nevertheless, the proposed mechanisms for the regulation of Rac1 are different, which might be due to each TRP channel’s differential interacting partners (Figure 2). A combination of complementary approaches to obtain a more global picture of the TRP channels-Rho GTPases network is needed to address this. The following section proposes some strategies to understand better the relationship between TRP ion channels and Rho GTPases.

Signaling mechanisms, such as those proposed above, often involve transient PPIs among regulatory components, which might represent a challenge for their identification due to the limited interaction time or low affinity of each interacting partners (Westermarck et al., 2013). Multiple proteomic approaches have emerged as alternatives to overcome these limitations (Bludau and Aebersold, 2020). These novel approaches could provide new possibilities to identify TRP/Rho GTPases PPIs pathways and give hints of the microdomains where these complexes localize.

Rho GTPases regulation by GEF/GAP/GDI is far more complex than a single GEF or GAP regulating only one Rho GTPase. Promiscuous activity has been reported for GEFs and GAPs, modulating more than one Rho GTPase (Bagci et al., 2020), increasing the complexity of the TRP-dependent modulation of these proteins. Proteomic studies may allow identification of GEFs/GAPs and GDIs implicated in Rho GTPases regulation by TRP channels. Complementing these approaches with assays for describing spatiotemporal dynamics will grant a more accurate characterization of the mechanisms involved in the TRP-dependent regulation of Rho GTPases and their role in neuropathological disorders. This will ultimately contribute to the designing of potential new interventions based on the TRP modulation of Rho GTPases. For instance, diverse strategies have been designed to monitor Rho GTPases activity through different means, such as biochemical approaches (Ren et al., 1999) and Förster Resonance Energy Transfer (FRET)-based sensors. The latter provides the possibility of performing *in vivo* tracking of GTPase activity (Kraynov et al., 2000; Pertz et al., 2006) and several authors have reviewed and corroborated its usefulness (Pertz, 2010; Schaefer et al., 2014). Recently, novel techniques to manipulate Rho GTPases have been designed. For instance, light-inducible sensors which promote RhoA (Wagner and Glotzer, 2016; Oakes et al., 2017) and Rac1 (Wu et al., 2009) translocation to the plasma membrane and subsequent activation. These tools might provide local and temporal inducible activation of Rho GTPases. Importantly, the application of these tools to the structures of TRP channels might also serve to define the role of these channels in the local and temporal regulation of Rho GTPases.

Another critical issue to study Rho GTPases regulation by TRP channels is the identification of Ca^2+^ as an intermediary in these pathways. Since local and broad Ca^2+^ waves have a differential effect on the modulation of Rho GTPases, the establishment of the spatiotemporal features of Ca^2+^ dynamics is necessary to strengthen what we know about these mechanisms. In this regard, fluorescent Ca^2+^ indicators derived from organic compounds provide information about the time-scale properties of Ca^2+^ waves but lack in providing accurate spatial information (Russell, 2011). Accordingly, genetically-encoded Ca^2+^ sensors provide an opportunity to study the time-scale and spatial distribution of Ca^2+^ signals, since they have been designed to monitor Ca^2+^ signals from intracellular organelles accurately (Bassett and Monteith, 2017). This approach has been used on neuronal models (Chen et al., 2012; O’Donnell et al., 2016), and even in the whole brain of living animals (Scott et al., 2018). These approaches, combined with biosensors to track the activity of Rho GTPases and its regulators, could provide novel and valuable information about the mechanisms involved in TRP channels-mediated regulation of Rho GTPases in physiological and pathophysiological brain conditions.

## Concluding Remarks

The study of neurological and neuropsychiatric diseases has been linked to tumoral growth, cell death and loss of cellular structures, such as dendritic spines and axonal processes. In this context, ion channels are essential in cell physiology, as the loss- or gain-of-function of these proteins trigger structural abnormalities and neuropathological conditions. TRP channels have emerged as novel candidates for the treatment of numerous CNS-affecting diseases. Thus, further knowledge of the mechanisms by which TRP channels exert their effects on cellular physiology constitutes an outstanding question. Herein, we exposed evidence and shared arguments and ideas that support general mechanisms by which TRP channels might modulate Rho GTPases in the brain. Although the evidence is strong enough to suggest several pathways, further studies are required to confirm the functional nature of these interactions. Future approaches considering quantitative proteomic analysis of TRP channels and Rho GTPases will reveal common partners that could mediate their functional relationship. Moreover, studies incorporating simultaneous analysis of localization, interaction, dynamic activity of Rho GTPases, TRP channels, and or GEFs/GAPs/GDIs will be required to determine the spatiotemporal features of these mechanisms. This new evidence would contribute to designing novel strategies based on the fine-tuning of transduction mechanisms involved in TRP-modulated processes, providing new therapeutic alternatives to overcome neuropathological conditions.

## Author Contributions

BL, PC, IS, OO-S, MPS, and OC wrote the manuscript. All the authors contributed to the article and approved the submitted version.

## Conflict of Interest

The authors declare that the research was conducted in the absence of any commercial or financial relationships that could be construed as a potential conflict of interest.
